# Acetic acid is an oxidative stressor in gastric cancer cells

**DOI:** 10.3164/jcbn.17-49

**Published:** 2018-04-03

**Authors:** Masahiko Terasaki, Hiromu Ito, Hiromi Kurokawa, Masato Tamura, Susumu Okabe, Hirofumi Matsui, Ichinosuke Hyodo

**Affiliations:** 1 Graduate School of Comprehensive Human Sciences, University of Tsukuba, 1-1-1 Tennohdai, Tsukuba, Ibaraki 305-8575, Japan; 2 General Corporative Association, Kyoto GI Disease Research Center, 671-1006 Marukizaimokucho, Nakagyo-ku, Kyoto 604-8106, Japan; 3 Kyoto Prefectural University of Medicine, 465 Kajii-cho, Kawaramachi-Hirokoji, Kamigyo-ku, Kyoto 602-8566, Japan

**Keywords:** acetic acid, reactive oxygen species, oxidative stress, gastric epithelial cell, apoptosis

## Abstract

Acetic acid can cause cellular injury. We previously reported that acetic acid induces cancer cell-selective death in rat gastric cells. However, the mechanism is unclear. Generally, cancer cells are more sensitive to reactive oxygen species than normal cells. Accordingly, in this study, we investigated the involvement of oxidative stress in cancer cell-selective death by acetic acid using normal gastric mucosal cells and cancerous gastric mucosal cells. The cancer cell-selective death was induced at the concentration of 2–5 µM acetic acid. Cancerous gastric mucosal cells had increased expression of monocarboxylic transporter 1 and high uptake of acetic acid, compared to normal gastric mucosal cells. The exposure of cancerous gastric mucosal cells to acetic acid enhanced production of reactive oxygen species and expression of monocarboxylic transporter 1, and induced apoptosis. In contrast, acetic acid showed minor effects in normal gastric mucosal cells. These results indicate that acetic acid induced cancer cell-selective death in gastric cells through a mechanism involving oxidative stress.

## Introduction

Acetic acid is a constituent of vinegar and has been used in human society for centuries. In ancient Greece, vinegar is used as a medicine for the treatment of sores and persistent cough.^(1)^ In addition, vinegar is currently used for protection of foods because of its preservative and bactericidal effects. In recent years, acetic acid has been reported to show some health benefits, including antihypertensive and anti-hyperglycemic effects.^(2,3)^ Some types of vinegar also show antitumor effects. Naturally fermented sugar cane vinegar (kibizu) from Amami Ohshima Island induces apoptosis in human leukemia cells.^(4)^ Additionally, vinegar produced by rice-*shochu* postdistillation slurry suppresses the growth of tumors and prolongs life span in mice bearing solid tumors.^(5)^

Vinegar contains roughly 5 vol% acetic acid, and the acetic acid in vinegar has been shown to have antitumor effects. Indeed, in a previous study, we reported that 0.5 vol% acetic acid induced cell death, particularly in cancer cells.^(6)^ In contrast, 60 vol% acetic acid causes cellular necrosis and ulcers following topical treatment.^(7)^ These data indicated that the cytotoxic effects of acetic acid depend on the concentration of the chemical. However, the mechanism through which acetic acid induces cell death has not been clarified.

Acetic acid is incorporated into cells via a membrane transporter, monocarboxylic transporter (MCT), which transports acetic acid or other monocarboxylic acids; acetic acid then becomes a substrate of acetyl-CoA and is used in the tricarboxylic acid (TCA) cycle.^(8)^ The acetic acid may induce oxidative stress and subsequent apoptosis in cancer cells. Reactive oxygen species (ROS), such as superoxide radicals, are produced through the TCA cycle, and the resulting ROS then induces apoptosis in cancer cells.^(9)^

In this study, we evaluated the cancer cell-selective toxic effects of acetic acid using a fluorescent co-culture model containing both normal and cancerous cells.^(10)^ Specifically, we evaluated rat gastric mucosal cells (RGM1 cells) and cancer-like cells (RGK1 cells) exposed to the carcinogenic agent of *N*-methyl-*N*’-nitro-*N*-nitrosoguanidine (MNNG).^(11)^ Our findings provide important insights into the mechanisms through which acetic acid affects cancer cells.

## Materials and Methods

### Materials

The Cell Counting Kit-8 and Diphenyl-1-pyrenylphosphine (DPPP) were purchased from Dojindo (Tokyo, Japan), anti-monocarboxylate transporter 1 (MCT1) antibodies were obtained from Alpha Diagnostic International, Inc. (San Antonio, TX), and anti-caspase 9 antibodies were obtained from Cell Signaling Technology, Inc. (Danvers, MA). Radioisotope of [2-^14^C]-acetic acid sodium salt was purchased from American Radiolabeled Chemicals, Inc. (St. Louis, MO). Acetic acid, hydrochloric acid and *N*-Acetyl-l-cysteine (NAC) were purchased from Wako Pure Chemical Industries, Ltd. (Osaka, Japan). The 12% Bis Tris gels were obtained from Life Technologies Japan, Ltd. (Tokyo, Japan), and the Can Get Signal reagents were obtained from TOYOBO Co., Ltd. (Osaka, Japan). 5,5-dimethyl-1-pyrroline-*N*-oxide (DMPO) was purchased from LABOTEC Co., Ltd. (Tokyo, Japan).

### Cell culture

Following four cell-lines were used: RGM1, RGM-GFP, RGK1 and RGK-KO. Both RGM-GFP and RGK-KO are fluorescent cells: the former was transfected a green fluorescent protein (GFP) gene and the latter was transfected a kusabira orange (KO) protein gene to emit fluorescence of GFP and KO, respectively. RGM1 and RGM-GFP cells were cultured in Dulbecco’s modified Eagle’s medium (DMEM)/F12 with l-glutamine (Life Technologies Japan, Ltd.) and RGK1 RGK-KO cells were DMEM/F12 without l-glutamine (Sigma-Aldrich Japan K.K., Tokyo, Japan). Culture media contained 10% inactivated fetal bovine serum (FBS; Biowest LLC, Kansas, MO) and 1% penicillin/streptomycin (Life Technologies). All cells were cultured in a cell culture incubator at 37°C in an atmosphere containing 5% CO_2_.

### Cell viability assay

Cell viability assays were performed using a Cell Counting Kit-8 according to the manufacturer’s instructions. RGM1 or RGK1 cells were cultivated on 96-well plates for 24 h at a concentration of 1 × 10^4^ cells/well. Culture medium was refreshed with medium containing 0, 1, 2, 5, 10, or 20 µM acetic acid, and cells were incubated at 37°C for 24 h. After incubation, the medium was replaced with medium containing 10% Cell Counting Kit-8 reagent. The absorbance was measured at 450 nm using a DTX880 multimode microplate reader (Beckman Coulter, Inc., Brea, CA) following incubation at 37°C for 1 h.

### Evaluation of cancer cell-selective toxicity

 Fluorescence-based co-cultures of normal and cancerous cells were used. The co-culture dishes were prepared at 31,250 cells/cm^2^ (15,625 cells/cm^2^ for each cell type). After incubation for 24 h, cells were exposed to hydrochloric acid or acetic acid at 0, 1, 2, 5, 10, or 20 µM for 24 h. Medium containing chemopreventive agents was then exchanged with fresh medium. Cells were observed each day using a confocal laser microscope until cells reached confluent (less than 240 h). The normal cell/cancer cell growth rate ratio was calculated as previously described.^(10)^

### Cellular uptake of acetic acid

Cellular uptake of acetic acid was examined using radioisotope-labeled acetic acid (acetic acid [2-^14^C] sodium salt: RI-acetic acid). Cells were cultured on 6-well plates at 1 × 10^5^ cells/well and incubated overnight. The medium was exchanged with fresh medium containing 5 µM RI-acetic acid and incubated at 37°C for 24 h. After incubation, cells were washed using PBS, RIPA buffer was added for cell lysis, and lysates were collected into vials containing liquid scintillation fluid (Pico-Fluor 40). The radiation doses of the samples were determined in a liquid scintillation counter (LSC-7200; Hitachi Aloka Medical, Ltd., Tokyo, Japan).

### Detection of lipid peroxidation

The lipid peroxidation was measured by DPPP. Cells were cultured on 4 chamber slide at 31,250 cells/cm^2^ and incubated overnight. Cells were exposed to 5 µM acetic acid or 5 µM acetic acid with 50 µM NAC for 24 h. The culture medium was replaced to the culture medium contained 10 µM DPPP. After incubated 15 min, cells were washed twice with PBS. The fluorescence intensities at Ex. 352 nm and Em. 380 nm of DPPP were measured by a plate reader (BZ-X710; Keyence Co., Tokyo, Japan).

### Western blotting of MCT1 and caspase 9

Western blotting was performed previously described.^(12,13)^ Briefly, 15 µl cell lysis solution (10 µg) from each cell type was prepared with NuPAGE LDS Sample buffer containing sample reducing agent (Life Technologies Japan, Ltd.) and boiled at 70°C for 10 min. For sodium dodecyl sulfate polyacrylamide gel electrophoresis (SDS-PAGE), the cell lysates were loaded into the wells of NuPAGE Novex 12% Bis-Tris gels. Gels were electrophoresed at 200 V for 30 min, and proteins were transferred onto polyvinylidene difluoride (PVDF) membranes (Millipore Co., Burlington, MA) by electrophoresis at 2.0 mA/cm^2^ for 60 min. The sandwich immunoassay was performed by Trans-Blot (Bio-Rad Laboratories, Inc., Hercules, CA), a suction-type immunoreaction system; antibody reaction steps were performed for about 30 min. Membranes were exposed to 15 ml of PVDF Blocking Reagent for Can Get Signal, and anti-rat MCT-1 antibodies (Alpha Diagnostic Intl. Inc., San Antonio, TX) or anti-rat caspase 9 antibodies (Cell Signaling Technology, Inc., Danvers, MA) were added to Can Get Signal Immunoreaction Enhancer Solution 1; the membranes were then incubated with the solution for 60 min. After the primary antibody solution was aspirated, the membrane was washed three times with 15 ml PBST (phosphate-buffered saline [PBS; Wako] plus 0.1% Tween-20). Horseradish peroxidase (HRP)-linked anti-rabbit IgG antibodies were added to Can Get Signal immunoreaction enhancer solution 2, and membranes were then treated with the solution for 60 min. The membranes were finally immersed in Lumina Forte Western HRP Substrate (Millipore), and luminescence was determined using an ImageQuant LAS 4000 (GE Health Care Japan, Tokyo, Japan). β-Actin was measured as a control for protein loading.

### Electron spin resonance (ESR) measurement with living cells

Intracellular ROS was measured by ESR using living cells, as previously reported.^(14)^ Cells were cultured on slide glass until confluent. The slide glass was immersed in acetic acid-containing medium (5 µM) for 60 min in a 5% CO_2_ incubator at 37°C. After incubation, the slide glass was placed on the tissue glass (Radical Research Inc., Tokyo, Japan). Eighty microliters of the solution for ESR measurement, which was prepared by dissolving respiratory substrates (5 mM succinic acid, 5 mM malic acid, 5 mM d-glutamic acid, and 5 mM NADH) and 5.9% v/v DMPO, was poured onto the tissue glass. The ESR spectra were then recorded using a JEOL-TE X-band spectrometer (JEOL, Tokyo, Japan). All ESR spectra were obtained under the following conditions: 10 mW incident microwave power, 0.1 mT modulation width, 8 min sweep time, 7.5 mT sweep width, 0.1 s time contrast, 333.5 mT center field, and 15 mT scan range. Spectral computer simulations were performed using a Win-Rad Radical Analyzer System (Radical Research).

### Statistical analysis

Static significant value (*p* value) was calculated using SPSS software (IBM Corp., Armonk, NY) followed by Tukey HSD.

## Results

### Acetic acid induced cancer cell-selective toxicity

**Fig. 1:**
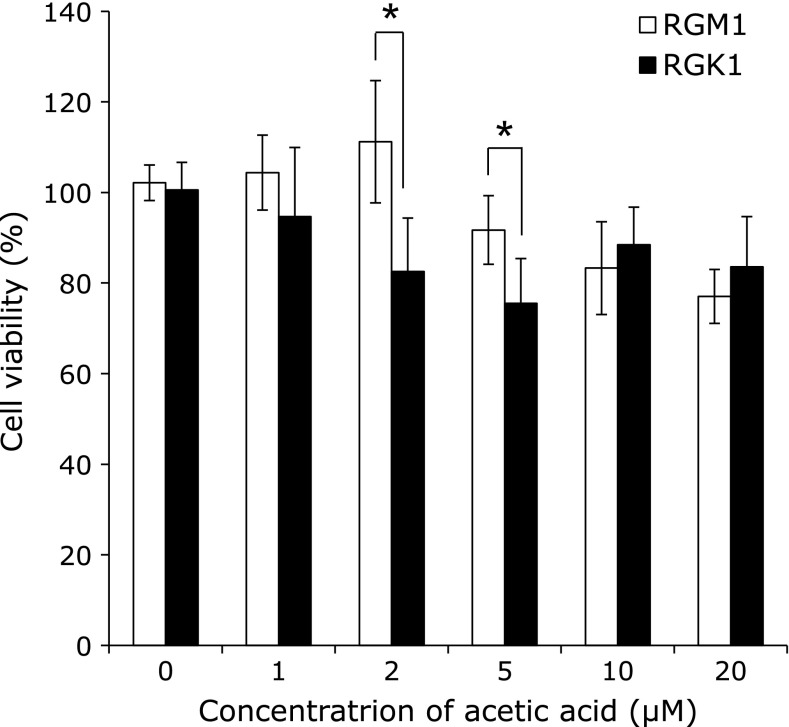
Cell viability test after acetic acid treatment. RGM1 and RGK1 cells were treated with acetic acid at concentrations of 0–20 µM for 24 h. *p<0.05. Error bars indicate SD (*n* = 4).

 First, we determined the viability of RGM1 and RGK1 cells exposed to acetic acid. Treatment with 2 or 5 µM acetic acid induced a greater degree of cell death in RGK1 cells than in RGM1 cells to show a significant difference (Fig. 1). Cancer cell-selective toxicity was not observed at other concentrations.

### Acetic acid induced cancer cell-selective toxicity in co-cultures of normal and cancer cells

**Fig. 2:**
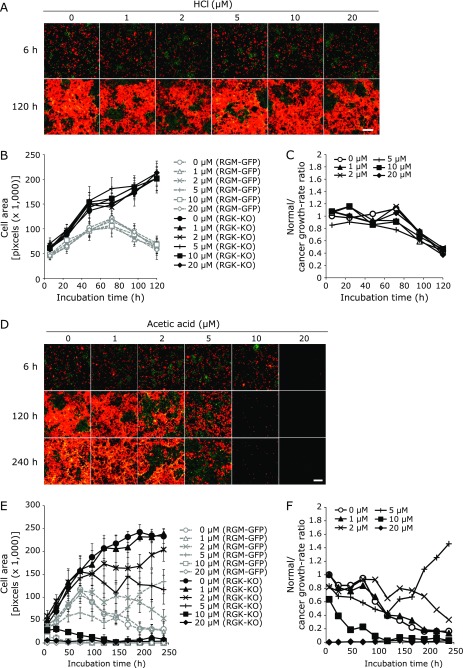
Cell viability assay following co-culture of RGM-GFP and RGK-KO cells. Time indicates the time after hydrochloric acid or acetic acid treatment for 24 h. Fluorescence images after hydrochloric acid treatment (A). Red and green fluorescence show RGM-GFP and RGM-KO cells, respectively. Scale bar: 500 µm. (B) Cell growth over time after hydrochloric acid treatment for 24 h. Error bars indicate SD (*n* = 6). (C) The normal cell/cancer cell growth rate ratios after hydrochloric acid treatment. (D) Fluorescence images after acetic acid treatment. Scale bar: 500 µm. (E) Cell growth over time after acetic acid treatment for 24 h. Error bars indicate SD (*n* = 6). (F) The normal cell/cancer cell growth rate ratios after acetic acid treatment.

Next, we determined the cancer cell-selective toxicity of acetic acid with a co-culture system using a couple of fluorescent cells; normal (RGM-GFP) and cancer cells (RGK-KO). Acetic acid showed cytotoxic effects in a cancer cell-specific manner; however, HCl did not show such effects (Fig. 2). Under HCl treatment, the cell areas of RGM-GFP and RGK-KO cells increased with time; however, the area of RGM1 decreased after 72 h (Fig. 2B). The normal cell/cancer cell growth rate ratio also decreased over time (Fig. 2C). In contrast, 2 and 5 µM acetic acid inhibited tumor cell growth while RGM-GFP cells continued growing up (Fig. 2D and E). When used at a concentration of less than one µM, acetic acid did not have any dramatic effects, and more than 10 µM caused cell death within 48 h (Fig. 2E). The normal cell/cancer cell growth rate ratio increased at 120 h following exposure to 5 µM acetic acid, and this increase occurred only at 5 µM, not at 2 µM or other concentrations (Fig. 2F). Thus, 5 µM acetic acid was the best concentration for cancer cell-selective toxicity.

### Cancer cell-specific uptake of acetic acid

**Fig. 3:**
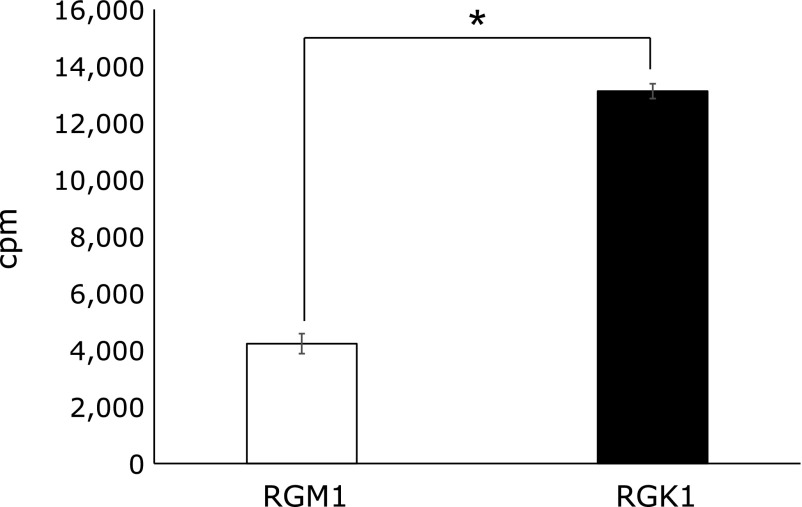
Cellular uptake of RI-labeled acetic acid. 5 µM RI-acetic acid was exposed for 24 h. Error bars indicate SD (*n* = 6). *p<0.05.

**Fig. 4:**
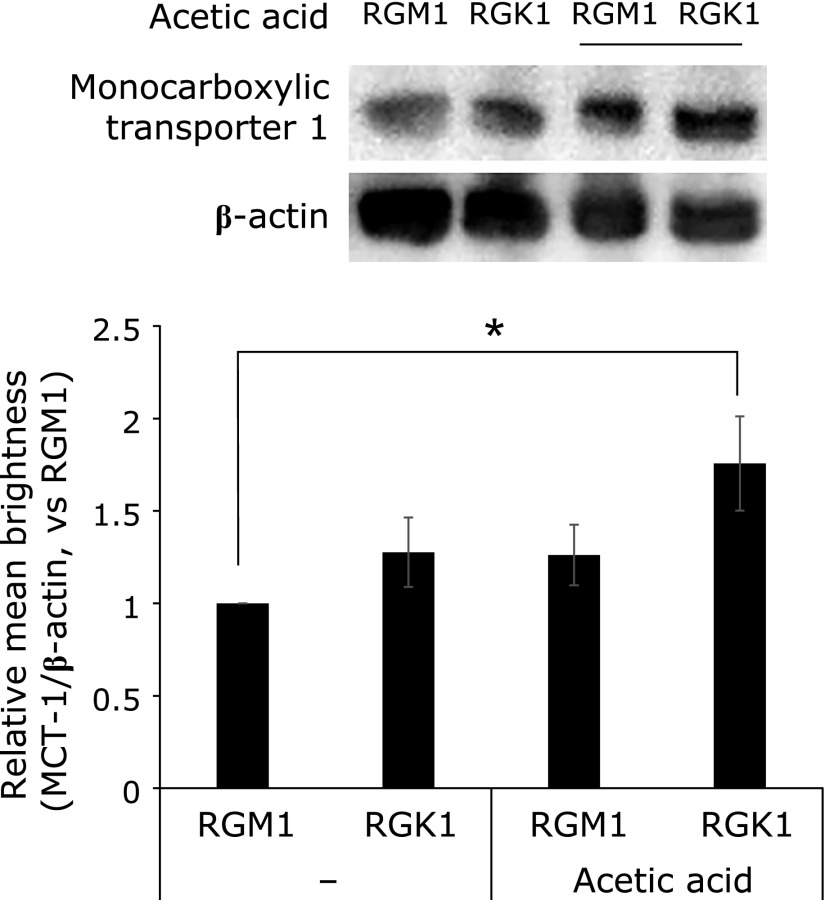
Measurement of MCT1 expression by western blotting. RGM1 and RGK1 cells were stimulated with 5 µM acetic acid for 24 h. Relative mean brightness was measured by ImageJ. Error bars indicate SD (*n* = 3). *p<0.05.

Next, we measured the cellular uptake of acetic acid to confirm cancer cell-selective toxicity using RI-labeled acetic acid (^14^C-acetic acid). The cellular uptake of acetic acid by RGK1 cells was approximately three times greater than that by RGM1 cells (Fig. 3). Additionally, MCT1 expression was increased following exposure to acetic acid, and tended to higher in RGK1 cells compared with RGM1 cells (Fig. 4). These results suggested that acetic acid accumulated in cells by inducing MCT1 expression.

### Acetic acid induced cancer cell-selective apoptosis

**Fig. 5:**
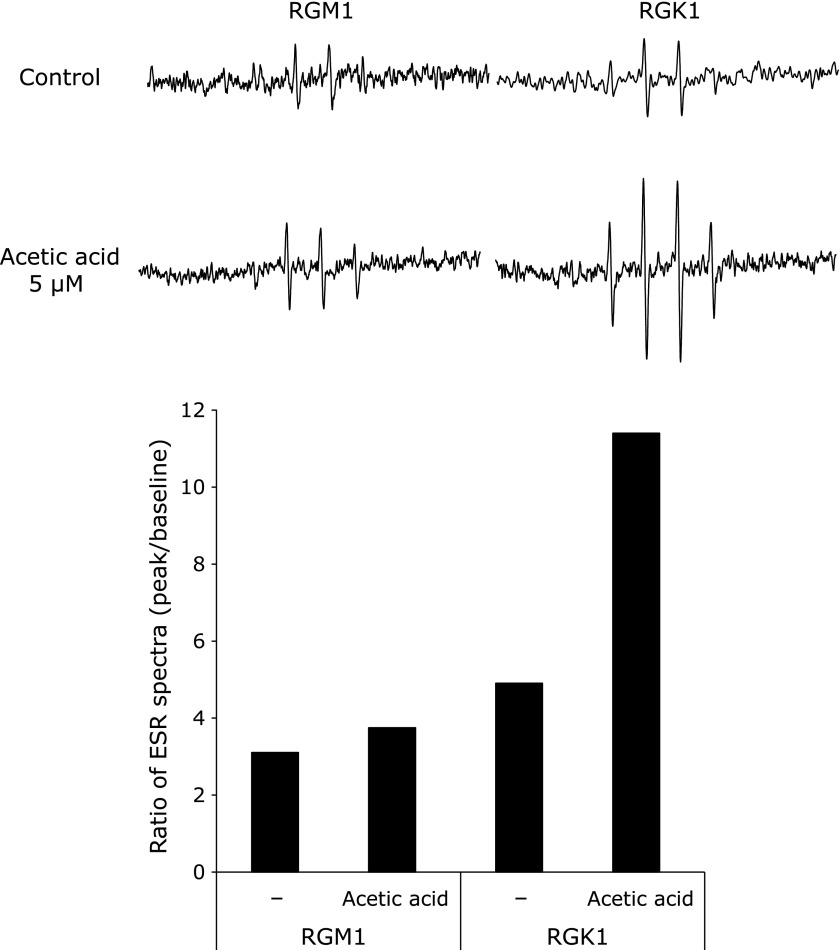
Measurement of cellular ROS in RGM1 and RGK1 cells by ESR. RGM1 and RGK1 cells were stimulated with 5 µM acetic acid for 60 min. The ratio of ESR spectra was shown in below (*n* = 1). DMPO was used as a spin-trapping reagent.

**Fig. 6:**
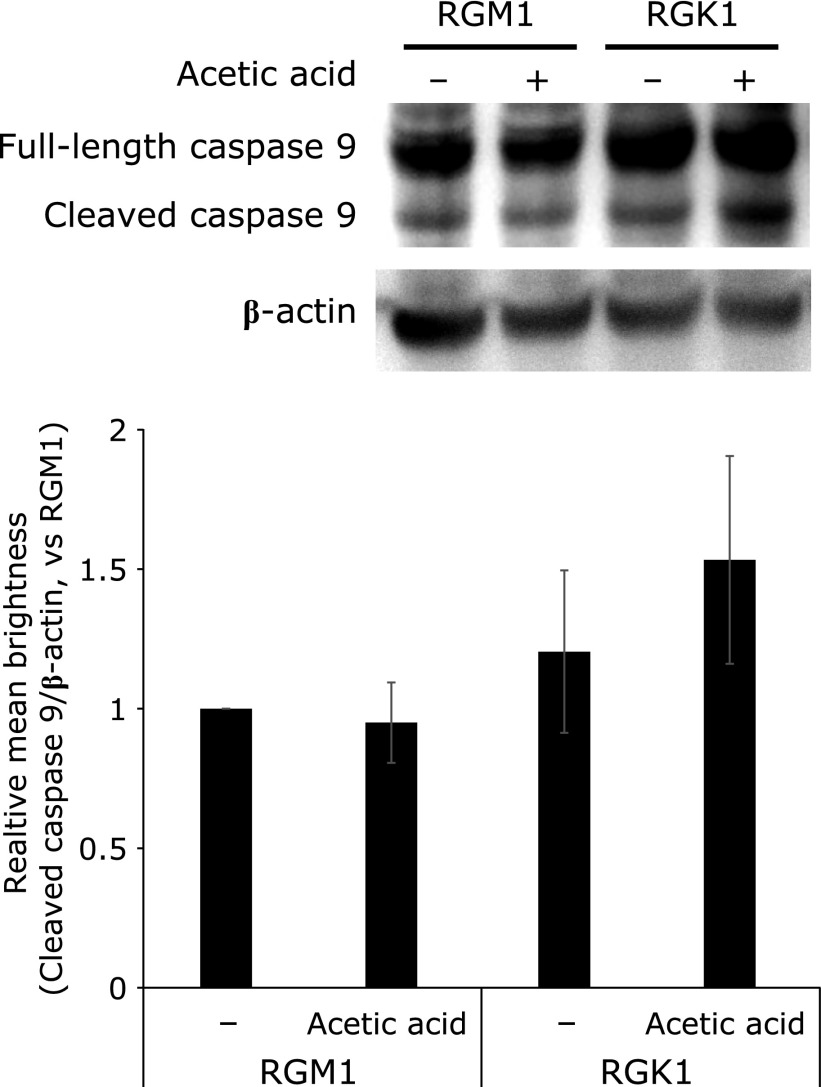
Measurement of caspase 9 expression by western blotting. RGM1 and RGK1 cells were stimulated with 5 µM acetic acid for 24 h. Relative mean brightness was measured by ImageJ. Error bars indicate SD (*n* = 3).

 The amounts of ROS were measured by ESR using living cells. The intensity of the ESR signal was increased by exposure to acetic acid in both RGM1 and RGK1 cells (Fig. 5). Interestingly, ROS levels were higher in RGK1 cells than in RGM1 cells and were increased following acetic acid treatment, thereby indicating that acetic acid induced cellular ROS production. Additionally, the expression of cleaved caspase 9 showed a tendency of an increase in acetic acid-treated RGK1 cells compared with that in acetic acid-treated RGM1 cells (Fig. 6). These results indicated that acetic acid induced cancer cell-selective apoptosis by stimulating cellular ROS production.

### Acetic acid induced cellular lipid peroxidation

**Fig. 7:**
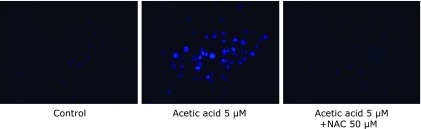
The acetic acid-induced lipid peroxidation was measured with DPPP-oxide fluorescence. RGK1 was stimulated with 5 µM acetic acid or 50 µM NAC with 5 µM acetic acid for 24 h.

For determining the oxidative stress by acetic acid, DPPP assay revealed that the fluorescence intensity of DPPP-oxide of acetic acid group was increased and was significantly different from that of control group (Fig. 7). Furthermore, the fluorescence was suppressed by NAC. This result indicated that acetic acid induced lipid peroxidation as a result of oxidative stress.

## Discussion

In this study, we demonstrated that acetic acid induced cancer cell-selective toxicity, likely through a pathway involving oxidative stress. In the present study, 5 µM acetic acid caused cancer cell-selective toxicity in co-cultures of RGM-GFP and RGK-KO cells. Moreover, acetic acid accumulated to a greater extent in RGK1 cells than in RGM1 cells, and MCT1 expression was increased after treatment with acetic acid. Finally, acetic acid stimulated ROS production in RGM1 and RGK1 cells and resulted in enhancement of cleaved caspase 9 levels, particularly in RGK1 cells. These results indicated that cancer cell-selective toxicity was caused by differences in the cellular uptake of acetic acid and cellular ROS concentrations between RGM1 and RGK1 cells.

In our previous study, we reported that a pH of 3 or 4 induces generation of ROS via mitochondrial dysfunction and subsequent apoptosis.^(15)^ Notably, 0.1% and 0.01% acetic acid in culture medium results in pH values of 6.8 and 7.4, respectively.^(6)^ Additionally, 5 µM acetic acid is lower than 0.01%, and the pH of the solution remains approximately 7.4. Thus, the cytotoxic effects of acetic acid are not related to pH.

Cancer cell metabolism involves the use of anaerobic glycolysis, even in an aerobic environment, a phenomenon known as the Warburg effect.^(16,17)^ If acetic acid induces oxidative stress in cancer cells through aerobic metabolism, cancer cell-selective toxicity in this study could be explained by cellular metabolism. That is, acetate is an important source of acetyl-CoA for the synthesis of fatty acids in hypoxic cancer cells.^(8)^ Moreover, ^11^C-acetate positron emission tomography is useful for detecting cancer cells because of cancer-specific acetic acid uptake.^(18)^ Furthermore, dichloroacetate (DCA), which inhibits pyruvate dehydrogenase kinase, shifts metabolism from cancer-specific glycolysis to oxidative phosphorylation and induces cancer cell apoptosis.^(9)^ Therefore, the incorporated acetic acid in cancer cells is metabolized into acetyl-CoA, and oxidative phosphorylation occurs. Accordingly, cancer cells undergo apoptosis owing to oxidative stress. Thus, there are possibly different pathways for acetic acid uptake from normal cells, and production of ATP possibly causes cellular injury in the presence of certain concentrations of acetic acid owing to ROS production.

Cancer cells require acetic acid to survive under nutrient-limiting conditions.^(19)^ In this study, 5 µM acetic acid caused apoptosis in RGK1 cells and enhanced the growth of RGM1 cells after treatment for 120 h. Cells were almost confluent at the time, indicating hypoxic and nutrient-limiting conditions. The culture conditions changed following replacement with fresh medium at 120 h, and replacement of culture medium seemed to become a second trigger for acetic acid-induced cytotoxicity. Cancer cells may switch metabolism depending on the presence of acetic acid and the culture conditions. However, further studies are needed to fully elucidate these mechanisms.

Acetic acid accumulated in cancer cells more than normal cells and induced cancer cell-selective cytotoxicity by stimulating ROS production. The observed cytotoxicity was dependent on the concentration of acetic acid. Thus, our results provided important insights into the mechanisms through which acetic acid affected cancer cell-selective toxicity.
